# The Synthesis of Granular ZSM-23 Zeolite with a High Degree of Crystallinity and a Micro-Meso-Macroporous Structure, and Its Use in the Hydroisomerization of n-Hexadecane

**DOI:** 10.3390/nano14231897

**Published:** 2024-11-26

**Authors:** Olga S. Travkina, Dmitry V. Serebrennikov, Rezeda Z. Kuvatova, Alfira N. Khazipova, Nadezhda A. Filippova, Marat R. Agliullin, Boris I. Kutepov

**Affiliations:** 1 Institute of Petrochemistry and Catalysis, Ufa Federal Research Centre of the Russian Academy of Sciences (UFRC RAS), 450075 Ufa, Russiamaratradikovich@mail.ru (M.R.A.);; 2 Faculty of Chemical Engineering, Ufa State Petroleum Technological University, 450062 Ufa, Russia

**Keywords:** ZSM-23 zeolite, micro-mesoporous materials, catalysts for hydroisomerization of n-paraffins

## Abstract

This paper proposes a method for synthesizing granular ZSM-23 zeolite with a high degree of crystallinity and hierarchical porous structure. This method is based on crystallizing granules composed of powdered ZSM-23 zeolite and a specially prepared amorphous aluminosilicate. It has been shown that these granules have superior mechanical strength compared to granular zeolite-containing materials, which are made from a mixture of ZSM-23 zeolite crystals and Al_2_O_3_. It has been demonstrated that when 0.5% of Pt granular ZSM-23 zeolite is used, with a high degree of crystallinity and a hierarchical porous structure, it exhibits higher activity and selectivity in the hydroisomerization of n-hexadecane compared to a bifunctional catalyst, which is a mechanical mixture of ZSM-23 zeolite crystals and Al_2_O_3_, with the metal deposited on the granules.

## 1. Introduction

The most promising technology for producing low-freezing diesel fuels, biofuels, and base synthetic motor oils of Group III is catalytic isodewaxing [1]. This process is based on the selective hydroisomerization of the higher n-paraffins C_16+_ found in fuel or oil into isoparaffins using bifunctional catalysts. These catalysts contain an active phase, such as NiS, CoMoS, Pd, or Pt, which is applied to acidic molecular sieves that are granulated with a binder [2]. Among the most selective molecular sieves for this process are SAPO-11 and ZSM-23 [3,4]. There has also been recent work on SAPO-31 molecular sieves for the hydroisomerization of long-chain n-paraffins (i.e., n-hexadecane) [5]. Currently, in industrial processes for the hydroisomerization of n-paraffins, catalytic systems based on ZSM-23 zeolite are primarily used. These systems, due to their stronger Brønsted acid sites compared to SAPO molecular sieves, enable the catalytic process to occur at lower temperatures. Chevron and ExxonMobil have already developed several generations of isodewaxing catalysts based on ZSM-23 zeolite for the production of Group III synthetic base oils [6,7,8,9]. 

ZSM-23 zeolite is a member of the MTT structural type with a one-dimensional pore structure and a pore diameter of 5.4 Å [10]. This unique microporous structure allows for the conversion of n-paraffins into monomethyl- and dimethyl-substituted isomers, significantly reducing the contribution from hydrocracking, which is characteristic of paraffins with three or more methyl paraffins [11].

To further reduce the contribution of hydrocracking of higher n-paraffins, various methods have been developed for the synthesis of nanoscale and hierarchical ZSM-23 [12,13,14,15,16,17]. Unfortunately, most of these methods rely on crystal growth modifiers. The main drawbacks of these methods are their high cost and the reduced crystallinity of the resulting materials, which makes them unsuitable for use on an industrial scale.

In the industrial processes of isodewaxing fuels and oils, bifunctional catalytic systems based on ZSM-23 molecular sieves with binders are used [18,19,20]. Aluminum hydroxide with a boehmite structure is used primarily as a binder. During the heat treatment process, it transforms into γ-Al_2_O_3_. The content of the binder is at least 20% by weight. The resulting catalyst granules are a mechanical mixture of zeolite crystals and binding particles. At the same time, the activity of the granular ZSM-23 zeolite is reduced compared to that of the powdered form due to dilution and changes in the relative surface area, itself influenced by synthesis and heat treatment, possibly allowing pores in some zeolite crystals to become blocked by binding particles [18,19,21].

In [22], the creation of granular binder-free ZSM-5 zeolite was proposed. The method of synthesis is based on crystallizing a dried aluminosilicate gel into granules using sodium silicate as a temporary “binder”. The prepared granules are then crystallized at 180 °C for 24 h in a 0.01 M NaOH solution. It has been demonstrated that this method allows for the synthesis of granular binder-free ZSM-5 zeolite. However, it should be noted that this study does not provide information on the mechanical strength of granules, which is a crucial parameter for industrial catalysts.

A method of crystallization of dried aluminophosphate gels containing an ionic liquid and a secondary amine at 200 °C in solvent-free autoclaves was proposed for the synthesis of AlPO4-11 molecular sieves in the form of granules [23]. This approach allowed researchers to synthesize AlPO_4_-11 in the form of binder-free granules. In their work, they used an ionic liquid as the solvent and template, which makes this technology more complicated for industrial applications.

Therefore, the issues outlined above demonstrate the need for the development of a binder-free material based on ZSM-23, which has a hierarchical porous structure, i.e., a structure consisting of microporous (pore diameter < 2 nm), mesoporous (2–50 nm), and macroporous (>50 nm) [24], while also possessing high mechanical strength.

This work is dedicated to the method of synthesizing granular binder-free ZSM-23 zeolite with a hierarchical porous structure and its application in the hydroisomerization of higher n-paraffins.

## 2. Materials and Methods

### 2.1. Preparation of Powdered Zeolite

Powdered ZSM-23 zeolite with a Si/Al ratio = 38 was synthesized from a reaction gel with the following composition: 0.35Na_2_O*1.0SiO_2_*0.0125Al_2_O_3_*0.7DMF*30H_2_O. It was prepared using SiO_2_ sol (40% SiO_2_, Ludox AS-40, No. CAS 7631-86-9, Sigma-Aldrich, St Louis, MO, USA), aluminum nitrate (Al(NO_3_)_3_*9H_2_O, 98%, No. CAS 7784-27-2, Sigma-Aldrich, St. Louis, MO, USA) and dimethylformamide (DMF, 99%, No. CAS 68-12-2, Acros Organics, Morris Plains, NJ, USA).

The reaction gel was prepared by adding NaOH and distilled water to a calculated amount of 15 g SiO_2_ sol while stirring intensively. Then, the calculated amounts of the template and Al(NO_3_)_3_*9H_2_O were added to the resulting solution.

After mixing all the components, the prepared gels were aged at 60 °C for 24 h and then crystallized at 170 °C for 72 h. The resulting products were separated by centrifugation and washed with distilled water to achieve neutral pH. The powdered ZSM-23 zeolite, after drying, is designated as ZSM-23PW.

### 2.2. Preparation of Amorphous Aluminosilicate

An amorphous aluminosilicate with a Si/Al ratio of 39 was prepared by mixing 131.3 g sodium silicate (Na_2_SiO_3_, 99%, No. CAS 6834-92-0, Acros Organics, Geel, Belgium) and 61.3 g aluminum sulfate (Al_2_(SO_4_)_3_*18H_2_O, 99%, No. CAS 17927-65-0, Acros Organics, Geel, Belgium), dissolving in distilled water. The resulting mixture was then aged at 30 °C for 24 h. After that, the precipitate was filtered, washed with water, and dried at 120 °C for 5 h.

### 2.3. Preparation of Granular Samples

ZSM-23 zeolite granulated with amorphous aluminosilicate was prepared by mixing powdered ZSM-23 zeolite and amorphous aluminosilicate in a MX 0.4 mixer (VINCI Technologies, Nanterre, France) with the addition of polyvinyl alcohol plasticizer (99%, No. CAS 9002-89-5, Sigma Aldrich, Saint Louis, MO, USA).

Next, the resulting mixture was molded into granules (diameter of 1.4–1.5 mm, length of 5–6 mm) using an VTE1 extruder (VINCI Technologies Nanterre, France). Analogous to the synthesis of granular NaY zeolite with a hierarchical porous structure [25], the content of powdered ZSM-23 zeolite and binder in the granules was 60% and 40% by weight, respectively, based on dry zeolite. To prepare 200 g of the granulated sample, a mixture of 120 g of zeolite and 80 g of amorphous aluminosilicate was wetted to 53% with a 5 wt % aqueous solution of PVA. The resulting granules were dried in air at 30 °C for 24 h and then at 120 °C for 6 h. Then, granules were calcined at 600 °C for 4 h in order to remove the plasticizer that was contained in the granules. The calcined pellet samples were then designated as ZSM-23GR. To prepare highly crystalline granular ZSM-23, pellet ZSM-23GR was crystallized in an aqueous solution of sodium silicate with the addition of dimethylformamide (DMF) at 165 ± 5 °C for 24–32 h. The reaction mixture was composed of (0.25–0.36)Na_2_O∙(0.4–0.8)DMF∙(0.01–0.02)Al_2_O_3_∙SiO_2_∙(20–40)H_2_O. After crystallization, the granules were separated from the liquid phase, then washed from excess alkali (to pH ~ 8.0), and dried at 140–150 °C. The resulting granules are further designated as ZSM-23WB.

The granular acid carrier, a mechanical mixture of powdered ZSM-23PW zeolite and γ-Al_2_O_3_, was prepared as follows: powdered ZSM-23PW zeolite and boehmite (AlO(OH), 78% Al_2_O_3_, No. CAS 1318-23-6, Sasol, Hamburg, Germany) were thoroughly mixed to form a homogeneous mass (with a 20% content of boehmite by weight in terms of Al_2_O_3_), the resulting mixture was wetted with 5% nitric acid (HNO_3_, 67%, No. CAS 7697-37-2, Reachem, Moscow, Russia), and granules were molded using an extruder. Granules with a diameter of 1.4–1.5 mm and a length of 5–6 mm were dried at 100 °C for 24 h and then calcined at 600 °C for 6 h, during which time the boehmite converted to aluminum oxide. The obtained sample is designated as ZSM-23BD. The sample before calcination at 600 °C is designated as ZSM-23BDp.

The samples of ZSM-23 zeolite in H-form were prepared by ion exchange of Na^+^ for NH_4_^+^ in a 0.9 M aqueous solution of ammonium nitrate (NH_4_NO_3_, 98%, No. CAS 6484-52-2, Sigma Aldrich, Saint Louis, MO, USA) at 70–90 °C for 1 h with stirring. The solid-to-liquid ratio was 1/7 by weight. The samples were then dried at 120–150 °C and calcined at 540–550 °C for 4 h in an atmosphere. ZSM-23 samples in the H-form synthesized from ZSM-23PW, ZSM-23WB, and ZSM-23BD are designated as HZSM-23PW, HZSM-23WB, and HZSM-23BD, respectively.

### 2.4. Preparation of Bifunctional Catalytic Systems

From the samples HZSM-23WB and HZSM-23BD, a fraction between 0.1 and 0.5 mm was obtained by crushing and sifting through sieves. This material was then subjected to heat treatment at 350 °C for 6 h in an air atmosphere. After that, it was impregnated with an aqueous solution of H_2_PtCl_6_ × 6H_2_O (99%, No. CAS 26023-84-7, Sigma Aldrich, Darmstadt, Germany) (at a rate of 0.5 weight percent Pt per carrier weight), dried at 100 °C for 24 h, and calcined at 550 °C for 5 h.

Before the reaction, the catalyst fraction was reduced under a hydrogen flow at 400 °C for 5 h. The samples promoted with 0.5% Pt are designated as Pt/ZSM-23WB and Pt/ZSM-23BD, respectively.

### 2.5. Methods of Materials Analysis

The chemical compositions of the prepared amorphous aluminosilicate, zeolite powder, and granules were determined by energy-dispersive X-ray fluorescence spectroscopy using a Shimadzu EDX-7000P spectrometer (Shimadzu Corporation, Duisburg, Germany).

The phase compositions of the zeolites were analyzed using a Shimadzu XRD-7000 diffractometer (Shimadzu Corporation, Kyoto, Japan) with CuKα radiation. Scanning was performed in the range of angles 2θ from 3° to 40°, with increments of 1°/min. X-ray diffraction data were processed using Shimadzu XRD software (version 7.04) with the PDF2 database (version 2.2201) for phase analysis. Crystallinity was assessed using the content of the amorphous halo, which was measured in the range of 20 to 30 degrees 2θ using Shimadzu XRD Cristallinity software (version 7.04). The degree of crystallinity was calculated as the ratio of the total integrated intensity from the crystalline phase to the total integrated intensity from the crystalline and amorphous phases.

The morphology and crystal size of the granular molecular sieves were determined using scanning electron microscopy (SEM) with field emission from a Hitachi Regulus SU8220 scanning electron microscope (Hitachi High-Tech Corporation, Tokyo, Japan). The images were taken in the secondary electron mode at an accelerating voltage of 5 kV.

The porous structure characteristics (BET specific surface area and volume of micro- and mesopores) were determined by low-temperature N_2_ adsorption−desorption on a Quantachrome Nova 1200e sorption meter (Quantachrome Instruments, Boynton Beach, FL, USA). The volume of micropores and mesopores in the zeolite samples was calculated using the t-plot method, while the pore size distribution was determined using the BJH (Barrett–Joyner–Halenda) model. The macropore volume of the granular zeolite was estimated by mercury porometry using a Micromeritics AutoPore V instrument (Micromeritics Instrument Corporation, Norcross, GA, USA).

The acid site types and concentrations were determined by IR spectroscopy after pyridine adsorption (IR-Py). IR spectra of the adsorbed pyridine were recorded using a Bruker Vertex-70V IR spectrometer (Bruker Optic GmbH, Ettlingen, Germany) with a resolution of 4 cm^−1^. The samples were precalcined at 450 °C for 2 h in a 10^−2^ Pa vacuum. Pyridine was adsorbed onto the molecular sieve samples at 150 °C for 30 min. Pyridine was desorbed at 150 °C, 250 °C, and 350 °C. The concentrations of Brønsted acid sites (BAS) and Lewis acid sites (LAS) were calculated by integrating the absorbance bands at 1545 cm^−1^ and 1454 cm^−1^, respectively, using a previously described method [26].

The mechanical strength of the granular samples was evaluated using the LinteL PC-21 (Neftekhimavtomatika, Ufa, Russia) apparatus under static conditions through the compression method in automatic mode. The strength of the granules (length of 5–6 mm and diameter of 1.4–1.5 mm) was evaluated according to the ASTM D6175 method [27].

### 2.6. Hydroisomerization of Hexadecane

The hydroisomerization of n-hexadecane (*н-*C_16_H_34_, 99%, No. CAS 544-76-3, Acros Organics, Morris Plains, NJ, USA) on zeolite-containing catalysts was investigated in a flow reactor at temperatures ranging from 240 to 320 °C and a pressure of 3.0 MPa, with a molar ratio H_2_/*н-*C_16_H_34_ = 10, and a mass flow rate of 2 h^−1^. The liquid products of the catalytic transformation of n-hexadecane were identified using chromatographic mass spectrometry on a Shimadzu GCMS-QP2010 Ultra, while the gaseous products were analyzed using gas-liquid chromatography on a Chromatek-Crystal 5000 chromatograph (Chromatec, Yoshkar-Ola, Russia) with a flame ionization detector and a glass capillary column of 50 m.

## 3. Results

**Figure 1:**
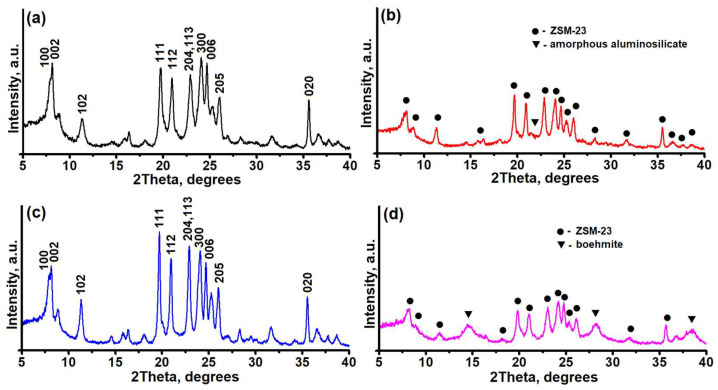
X-ray diffraction patterns of granular and powdered ZSM-23 zeolite samples: (**a**)—ZSM-23PW, (**b**)—ZSM-23GR, (**c**)—ZSM-23WB, and (**d**)—ZSM-23BDp.

It is known that the catalytic properties of molecular sieves depend significantly on the degree of crystallinity and the content of impurities from foreign phases. Figure 1 shows the results of the XRD analysis of the crystal structure of the synthesized samples.

It can be seen that all the diffractograms show peaks at the following angles: 7.88°, 8.2°, 8.9°, 11.4°, 19.8°, 21.0°, 23.0°, 24.8°, and 26.1°. These peaks are characteristic of the MTT type of zeolite [28]. Thus, the ZSM-23PW sample is a high-purity zeolite with a crystallinity of at least 95% (Figure 1a). ZSM-23GR granules are composite materials with a crystallinity degree of no more than 60%, consisting of ZSM-23 zeolite and an amorphous binder (Figure 1b). The data on the crystallinity of ZSM-23GR are in good agreement with the mass content of amorphous aluminosilicate in it. ZSM-23WB granules are a highly crystalline form of ZSM-23 zeolite with a degree of crystallinity of at least 92% (Figure 1c). It can be seen that the granules after crystallization have a similar level of crystallinity to that of the original powdered sample. This indicates a nearly complete transformation of the temporary binder (amorphous aluminosilicate) into ZSM-23 zeolite. The introduction of a boehmite-based binder into the granules (ZSM-23BDp sample) results in the appearance of additional 2ϴ peaks at 14.2°, 28.2°, 38.3°, 49.0°, and 72.0°, which are characteristic of boehmite AlO(OH) (Figure 1d). After calcination of ZSM-23BDp at 600 °C, the boehmite peaks disappear, and 2ϴ peaks of gamma aluminum oxide appear at 45.7° and 67.0° (XRD pattern is not shown in Figure 1).

**Figure 2:**
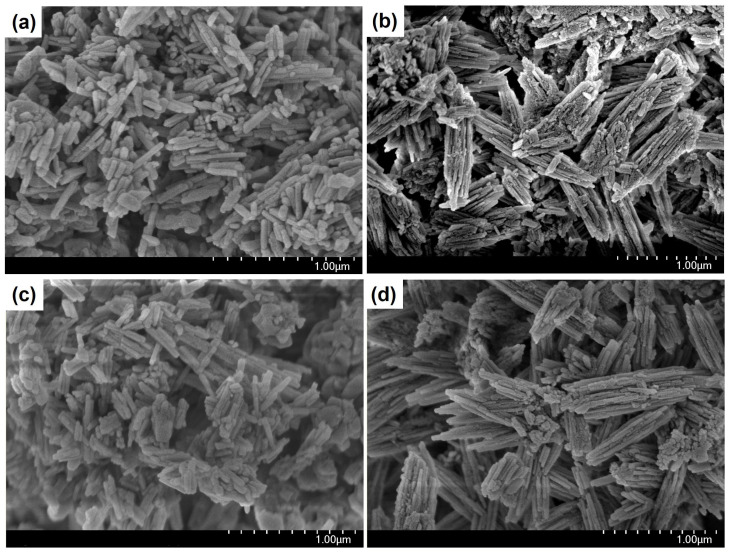
SEM images of granular and powdered ZSM-23 zeolite samples: (**a**) ZSM-23PW, (**b**) ZSM-23GR, (**c**) ZSM-23WB, and (**d**) ZSM-23BD.

The rate of diffusion in zeolite channels strongly depends on the morphology and size of the molecular sieve crystals [29]. Figure 2 shows the SEM images of the powdered ZSM-23 molecular sieve and its granules.

The ZSM-23PW sample consists of crystals in the form of elongated prisms, with a length of 200–300 nm and a thickness of approximately 50 nm. ZSM-23GR granules are composite materials composed of crystals of the ZSM-23 molecular sieve, which are in the form of elongated prisms with a crystal size of 200–300 nm and highly dispersed particles of amorphous aluminosilicate that fill the space between these crystals.

The ZSM-23WB sample contains clusters of nanocrystals with various morphologies, ranging from cubes to elongated prisms with dimensions between 200 and 300 nm. Elongated prisms appear to be the initial powdered form of zeolite, which is a part of the granules before crystallization. Nanocrystals in the shape of cubes, on the other hand, are crystals formed from an amorphous aluminosilicate material. The ZSM-23BD sample is a composite material that consists of crystals of the ZSM-23 molecular sieve, with crystal sizes ranging from 300 to 500 nm, and highly dispersed aluminum oxide particles that fill the spaces between the crystals.

**Figure 3:**
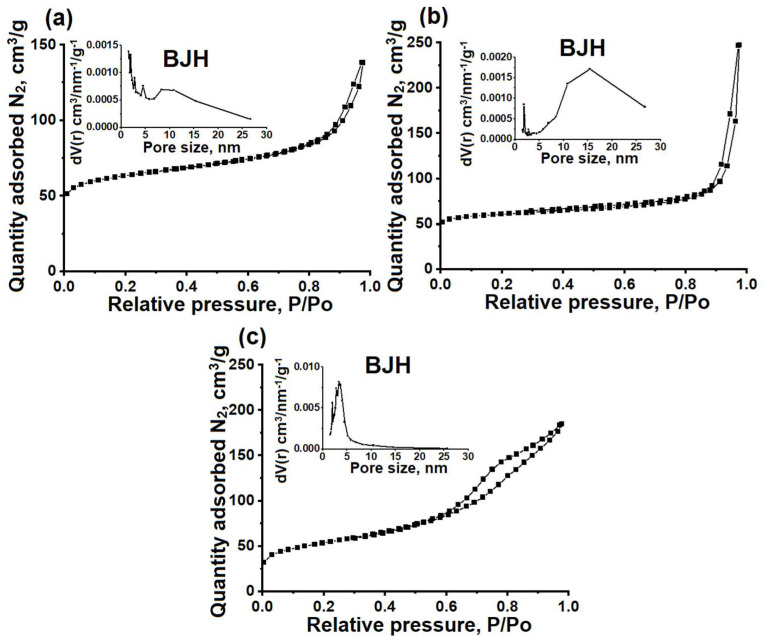
Nitrogen adsorption–desorption isotherms and pore size distribution (BJH) of granular and powdered ZSM-23 zeolite samples: (**a**) ZSM-23PW, (**b**) ZSM-23WB, and (**c**) ZSM-23BD.

As mentioned above, in order to effectively diffuse reacting molecules into zeolite micropores within granules, it is essential to create a well-developed secondary porous structure [28]. Figure 3 illustrates the nitrogen adsorption−desorption isotherms and pore size distribution, while Table 1 provides information on the characteristics of the porous structures of the ZSM-23PW, ZSM-23WB, and ZSM-23BD samples.

It can be seen that the studied samples exhibit isotherms that are close to type IV, with a sharp increase in the low-pressure region and a hysteresis loop with type H3 behavior in the pressure range between 0.8 and 1.0. This type of isotherm is typical for micro-mesoporous materials. The formation of mesopores in ZSM-23PW is attributed to the partial melting of the nanocrystals, as can be clearly seen from the SEM images in Figure 2.

The increase in the mesopore volume is attributed to the incorporation of mesoporous amorphous aluminosilicate into the composition. The ZSM-23WB sample has a higher specific surface area and mesopore volume compared to the ZSM-23PW sample. This can be explained by the formation of a secondary porous structure, which occurs due to the clustering of nanoscale crystals that are smaller than the original crystals of the ZSM-23 zeolite powder used to prepare the granules (Figure 2).

For the ZSM-23BD sample, the volume of micropores decreased by about 50%, and the specific volume of mesopores increased. These results can be explained by the dilution of the zeolite with a mesoporous binder, which partially blocked its micropores. According to the mercury porometry data, the ZSM-23WB sample has the largest volume of macropores among the granular samples. Therefore, granular ZSM-23WB is a hierarchical material with a well-developed secondary porous structure consisting of meso- and macropores.

Industrial hydroisomerization catalysts are granular materials that require a high level of mechanical crushing strength, as the upper layers of the catalyst can collapse under the weight of large loads in the reactor, damaging the lower layers. Table 2 presents the results of the study on the mechanical strength of the ZSM-23 granular samples.

It can be seen that after crystallization, the mean mechanical strength of the granules increased threefold compared to the initial granules. This can be explained by the fusion of the crystals introduced into the granules with those formed from the binder. As we have previously shown, such granular materials consist of a single cluster of crystals [25]. A comparison of the mean strength of the ZSM-23WB and ZSM-23BD samples shows that the hierarchical one is ~20% stronger due to these features of the formation of these granules. Student’s t-test showed that the differences are highly statistically significant (*p* < 0.05).

**Figure 4:**
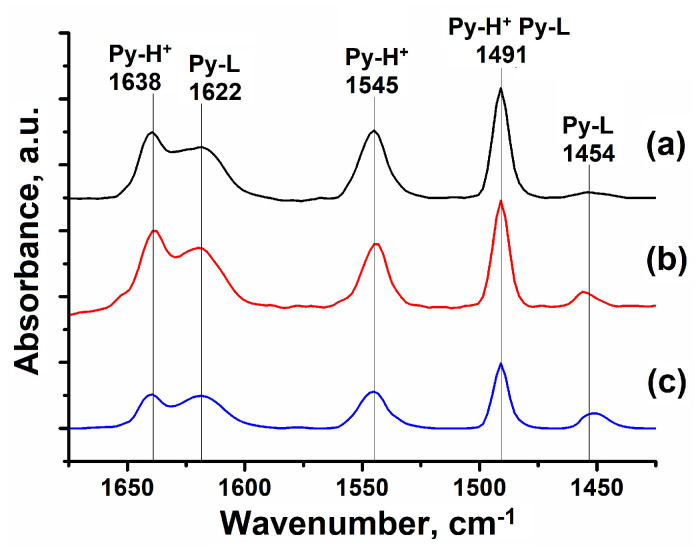
IR spectra of adsorbed pyridine for granular and powdered ZSM-23 zeolite samples: (**a**) HZSM-23PW, (**b**) HZSM-23WB, and (**c**) HZSM-23BD.

The catalytic properties of ZSM-23 molecular sieves depend strongly on their acidic properties. Using infrared spectroscopy of adsorbed pyridine, it has been shown that zeolite samples of ZSM-23 in the H-form contain two types of acidic sites: Brønsted acid sites (BAS) and Lewis acid sites (LAS) (Figure 4).

The oscillation bands at 1639 and 1545 cm^−1^ correspond to the protonated pyridine from the BAS. The bands at 1624 and 1454 cm^−1^ are attributed to pyridine, which has formed a coordination bond with LAS. The band at 1491 cm^−1^ is associated with both types of acid sites. Samples HZSM-23BD, which contain a binder in their composition, have the lowest levels of BAS and LAS (Table 3).

The results obtained, as shown above, can be explained by the dilution of zeolite with a binder, which blocks the one-dimensional channels of the ZSM-23 zeolite. This results in the acid sites becoming inaccessible to pyridine molecules. The LAS increases because aluminum oxide itself contains some Lewis acid centers. The HZSM-23PW and HZSM-23WB samples have the highest concentration of acidic sites because they do not contain any binders in their composition.

Thus, it can be seen that the HZSM-23WB granular material is characterized by significantly higher concentrations of acid sites due to the lack of a binder in its composition and the presence of smaller nano-sized crystals, which make most of the acid sites accessible.

It has been shown in [30] that when molecular sieves with a one-dimensional channel porous structure and 10R rings of platinum are impregnated with moisture in amounts of at least 0.5 wt %, a high dispersion of platinum is achieved, resulting in a high content of hydrogenating/dehydrating active sites.

In this case, the rate-limiting step in hydroisomerization through a bifunctional mechanism is primarily the isomerization step at the acidic sites of the molecular sieves.

**Figure 5:**
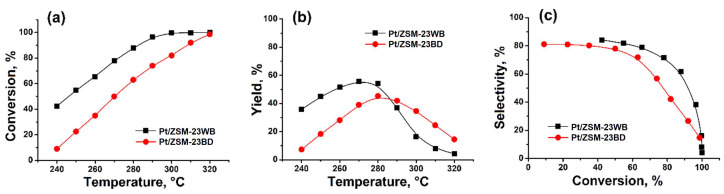
Hydroisomerization of n-hexadecane over 0.5% wt.% Pt/ZSM-23 samples: (**a**)—n-hexadecane conversion versus reaction temperature; (**b**)—C_16_ isomer yield versus reaction temperature; (**c**)—C_16_ isomer selectivity versus n-hexadecane conversion.

A comparison of the catalytic properties of ZSM-23 granular samples with binder and binder-free in the hydroisomerization of n-hexadecane revealed (Figure 5) that the granular sample binder-free exhibited higher activity: the conversion of n-C_16_ at 280 °C was 87.8%, while on the sample with a binder, it was 63.1% (Figure 5a). A higher yield of C_16_ isomers is achieved on the sample ZSM-23 zeolite binder-free: 55.8% vs. 45.3% at 270–280 °C (Figure 5b). The Pt/ZSM-23WB sample is more active and selective in the hydroisomerization of n-C_16_ compared to Pt/ZSM-23BD due to the higher content of the active component in this granular sample. At 63.1–65.5% conversion of hexadecane, the C_16_ isomer selectivities were 71.9 and 77.8 for ZSM-23BD and ZSM-23WB, respectively (Figure 5c). This means that the concentration of acid sites (Table 3) is noticeably higher, while the specific surface area and microporosity are still preserved (Table 1). In addition, the binder-free granules contain nanocrystals, which ensure good accessibility to acid sites.

The results obtained from the hydroisomerization of n-hexadecane suggest that in order to develop more efficient and selective granular catalysts based on ZSM-23 for the conversion of higher n-paraffins, it is essential to create materials that contain a minimum amount of binders. These materials must also have sufficient mechanical strength to ensure the catalyst’s operation for at least one year without degradation.

## 4. Conclusions

The crystallization of granules consisting of 60% by weight of powdered ZSM-23 and 40% by weight of a binder material, which is a dry amorphous aluminosilicate, was investigated using a variety of techniques: XRD, XRF, SEM, IR-Py, low-temperature nitrogen adsorption−desorption, mercury porosimetry, and IR spectroscopy with pyridine adsorption.

Based on the results obtained, we have developed a method for producing a granular ZSM-23 molecular sieve (d = 1.4–1.5 mm, l = 5–6 mm) with a hierarchical porous structure and high phase purity. The degree of crystallinity of the material is more than 92%. It allows obtaining a material with a specific surface area of 210 m^2^/g, a micropore volume of 0.06 cm^3^/g, a mesopore volume of 0.11 cm^3^/g, and a macropore volume of 0.25 cm^3^/g.

It was found that when applying 0.5% Pt by weight, a bifunctional catalyst is formed on the H-form of ZSM-23 zeolite, which is a highly crystalline and hierarchical porous structure. This catalyst exhibited higher activity and selectivity for the hydroisomerization of n-hexadecane than a catalyst prepared using granular ZSM-23 zeolite without a binder.

## Figures and Tables

**Table 1:** Characteristics of the porous structure in granular and powdered ZSM-23 zeolite samples.

Sample	S_BET_, m^2^/g	V_micro_, cm^3^/g	V_meso_, cm^3^/g	^Hg^V_macro_, cm^3^/g
ZSM-23PW	198	0.05	0.11	-
ZSM-23WB	210	0.06	0.25	0.40
ZSM-23BD	221	0.04	0.25	0.38

S_BET_—specific surface according to BET. V_micro_—specific volume of micropores. V_meso_—specific volume of mesopores. ^Hg^V_macro_—specific volume of macropores.

**Table 2:** Mechanical strength of granular ZSM-23 zeolite samples.

Sample	Mechanical Crushing Strength (Radial)		Mechanical Crushing Strength (Uniaxial), N/mm^2^
	N/pellet	N/mm^2^	
ZSM-23GR	18.2 ± 3.5	3.1 ± 0.5	3.2 ± 0.6
ZSM-23WB	57.1 ± 12.9	8.8 ± 2.1	9.8 ± 2.3
ZSM-23BD	44.3 ± 9.7	6.9 ± 1.5	7.9 ± 1.8

Results are shown as the mean ± standard deviation, SD (n = 24).

**Table 3:** Concentrations of acid sites in granular and powdered ZSM-23 zeolite samples according to IR spectroscopy data with pyridine adsorption.

Sample	Acidity (μmol/g)					
	BAS			LAS		
	150 °C	250 °C	350 °C	150 °C	250 °C	350 °C
HZSM-23PW	241	224	217	16	10	9
HZSM-23WB	232	221	203	23	17	14
HZSM-23BD	144	138	117	36	17	9

## Data Availability

The original contributions presented in the study are included in the article, further inquiries can be directed to the corresponding authors.
